# Cell cycle phase classification in 3D *in vivo* microscopy of *Drosophila* embryogenesis

**DOI:** 10.1186/1471-2105-12-S13-S18

**Published:** 2011-11-30

**Authors:** Tie Hua Du, Wee Choo Puah, Martin Wasser

**Affiliations:** 1 Imaging Informatics Division, Live-Cell Imaging and Automation of Image Analysis Group Bioinformatics Institute (BII), Agency for Science, Technology and Research(A*STAR), Singapore; 2 Department of Biological Sciences, National University of Singapore, Singapore

## Abstract

**Background:**

Cell divisions play critical roles in disease and development. The analysis of cell division phenotypes in high content image-based screening and time-lapse microscopy relies on automated nuclear segmentation and classification of cell cycle phases. Automated identification of the cell cycle phase helps biologists quantify the effect of genetic perturbations and drug treatments. Most existing studies have dealt with 2D images of cultured cells. Few, if any, studies have addressed the problem of cell cycle classification in 3D image stacks of intact tissues.

**Results:**

We developed a workflow for the automated cell cycle phase classification in 3D time-series image datasets of live *Drosophila* embryos expressing the chromatin marker histone-GFP. Upon image acquisition by laser scanning confocal microscopy and 3D nuclear segmentation, we extracted 3D intensity, shape and texture features from interphase nuclei and mitotic chromosomes. We trained different classifiers, including support vector machines (SVM) and neural networks, to distinguish between 5 cell cycles phases (Interphase and 4 mitotic phases) and achieved over 90% accuracy. As the different phases occur at different frequencies (58% of samples correspond to interphase), we devised a strategy to improve the identification of classes with low representation. To investigate which features are required for accurate classification, we performed feature reduction and selection. We were able to reduce the feature set from 42 to 9 without affecting classifier performance. We observed a dramatic decrease of classification performance when the training and testing samples were derived from two different developmental stages, the nuclear divisions of the syncytial blastoderm and the cell divisions during gastrulation. Combining samples from both developmental stages produced a more robust and accurate classifier.

**Conclusions:**

Our study demonstrates that automated cell cycle phase classification, besides 2D images of cultured cells, can also be applied to 3D images of live tissues. We could reduce the initial 3D feature set from 42 to 9 without compromising performance. Robust classifiers of intact animals need to be trained with samples from different developmental stages and cell types. Cell cycle classification in live animals can be used for automated phenotyping and to improve the performance of automated cell tracking.

## Background

**Figure 1:**
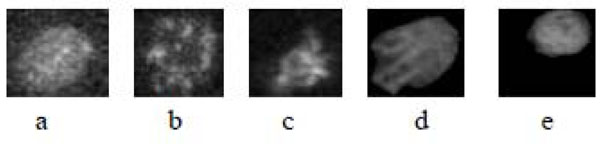
**Maximum intensity projections of nuclei in a live *Drosophila* embryo labelled with histone-GFP.** (a) Interphase, (b) prophase, (c) metaphase, (d) anaphase and (e) telophase

Cell divisions and their regulation play important roles in disease and development. The cell cycle can be divided in two main periods: interphase and mitosis. During interphase the cell grows, duplicates its DNA and accumulates nutrient and gene products required for mitosis. During mitosis, the cell splits itself and divides the genomic DNA between the two daughter cells. The mitosis can be further subdivided into several distinct phases: prophase, metaphase, anaphase and telophase. The cell phases can be identified by their appearance in high resolution microscopy images. Figure 1 shows examples of the typical appearances of the chromatin marker histone-GFP in different cell cycle phases. Automated cell phase classification is an essential step in high-throughput image analysis of large populations of cells that enables quantification of cell cycle progression, which is very important for developmental biology, cancer cell study and drug discovery. For instance, measuring the duration of individual cell cycle phases under different genetic and drug treatment conditions can improve the understanding of biological mechanisms in oncological diseases and enhance the effectiveness of drug discovery and development [1]. Cell phase classification is crucial for high-throughput image based screens, such as the Mitocheck project that are aimed at identifying and characterizing genes involved in cell division [2]. Several bioimaging research groups have addressed this challenging problem [3-7]. Most studies involved 2D images [1,3-6]. One study dealt with 3D images, but cellular features were extracted from the most informative single slice [7]. Dynamic features have been widely used for cell phase classification [1,5-7], however as mentioned by [1], tracking algorithms become less reliable and context information becomes less informative when the cells are densely populated or/and move at fast velocity. In recent years, confocal laser scanning microscopy (CLSM) has become a common imaging modality to visualize fluorescently labelled cells in 3D. The extra dimension compared to conventional 2D microscopy promises to enhance the understanding of bio-molecular mechanisms. Another application of automated cell cycle phase identification is the improvement of cell tracking in the analysis of time-lapse images. In live tissues, cells can move large distances. Significant displacements in short periods (e.g. one minute) are especially pronounced in mitosis of *Drosophila* embryos. Since cell cycle phases occur in a fixed order, tracking can be improved using this prior biological knowledge. Therefore, it is essential to develop a cell phase classification algorithm that utilizes 3D image information and does not rely on dynamic features extracted by cell tracking. In this article, we present an automated cell cycle phase classification algorithm for 3D images of live *Drosophila* embryos.

## Methods

### Microscopy

The images stacks of *Drosophila* embryo were recorded at 55-60 second time intervals using a Zeiss 5 Live confocal laser scanning inverted microscope and consisted of 66-70 slices of 1024 x 1024 pixels. The voxel dimensions in x/y/z were 0.1 x 0.1 x 0.44 microns.

### Image processing, segmentation and creation of labelled datasets

The image stacks were first deconvolved using Huygens Professional [8] to enhance the image quality. Then interphase nuclei and mitotic chromosomes were segmented using a multi-level-set 3D segmentation algorithm [9]. Data samples of nuclei were obtained from movies of two embryos. The first embryo was recorded during the syncytial blastoderm stage and gave rise to 4606 samples representing the 5 phases of nuclear division cycles (interphase, prophase, metaphase, anaphase, telophase). The second 3D time series image dataset was acquired after cellularization during the cell divisions of the gastrulation stage and gave rise to 3119 samples For each sample, we calculated a set of 42 3D features (see below) and assigned one of the five cell cycle labels.

### 3D feature calculation

Humans recognize objects by their geometric and photometric characteristics. To mimic human vision, a set of 42 3D shape, texture and intensity features was carefully designed and extracted.

#### Volume

The volume V is equal to the total number of voxels inside the object times the voxel size. V = n×sx×sy×sz.

#### Surface area

The surface area A is calculated using a voxel-based surface area estimation method [10]. Prior to surface area calculation, segmented image stacks were interpolated to make each voxel isotropic using a shape-based interpolation [11].

#### Sphericity

Humans tend to identify nuclei based on their round or spherical shape. Sphericity ψ is defined as .

#### Eccentricity

The eccentricity features E_1_, E_2_ are defined as the ratios of the square root of the third and second eigen value to the square root of the first eigen value. The inverse of the square root of the eigen values is the corresponding equatorial radius of an ellipsoid fitted into a given 3D object.

#### Mean and standard deviation of distance from surface to centroid

The voxels on the object surface are denoted as (p_1_,…,p_i_,…,p_m_), and their distances to the object centroid are (d_1_,…,d_i_,…,d_m_). The meanand standard deviation of surface to centroid distances are defined as  and .

#### Mean and standard deviation of intensity

Let the pixel intensities in 3D objects be denoted as (I_1_,…,I_i_,…,I_n_). The mean and standard deviation of intensity are defined as  and .

#### 3D texture features

Texture was described using Haralick texture features that are based on the 2D grey-level co-occurrence matrix (GLCM) [12-14]. In order to calculate 3D texture features, the grey-tone spatial dependence matrices P_k_(i,j)(k = 1,…,13,i = 1, …, 256,j = 1, …,256) are calculated in 13 instead of 4 directions. *N*_G_ denotes the number of grey levels, which is 256 in our case. Different displacement values of 1, 2, 4, and 8 were tested, all of which showed similar classification results. To reduce computational expenses and feature space dimensionality, we set the displacement value to 1 only.

The following texture features were used in this study:

Energy:

Entropy:

Correlation:

Where μ_x_, μ_y_, σ_x_ and σ_y_ are the means and standard deviations of p_x_ and p_y_.

Contrast:

Homogeneity:

Variance:

Sum entropy

Sum average

Sum variance

Difference entropy

Cluster shade

Cluster prominence

Difference variance

f_13_ = variance of p_x−y_

Max probability

Information measures of correlation 1

Information measures of correlation 2

Where HX and HY are the entropies of P_x_ and P_y_ and

For a given 3D object, we have 13 angular gray-tone spatial dependence matrices. Hence we obtain a set of 13 values for each of the above mentioned texture features. The mean and standard deviation of these 13 values served as the 3D texture features.

#### Deviation between intensity-weighted and geometrical centroids

The Geometrical centroid of a 3D object  is defined as . The intensity weighted-weighted centroid  is defined as . The Deviation between intensity-weighted centroid to geometrical centroid (dx, dy, dz) is defined as , which describes the intensity distribution within a 3D object. The motivation of this feature was to describe asymmetry of intensity distribution found in cells, such as condensed heterochromatin found at one end of an interphase nucleus.

### Feature reduction and classification

**Figure 2:**
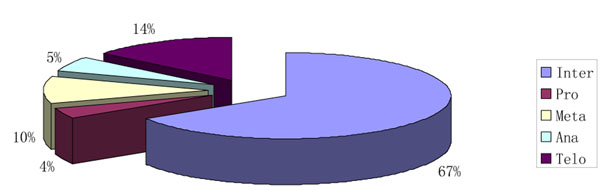
Imbalanced distribution of cell cycle phases.

We used a set of feature reduction and selection techniques to reduce the dimensionality of the feature space, including principle component analysis (PCA), linear discriminant analysis (LDA), Multidimensional scaling (MDS) [15-18], forward selection and backward elimination [19]. We tested several supervised machine learning methods to classify five different cell cycle phases, including the support vector machine (SVM) [20], probabilistic neural network (PNN), K-nearest neighbour (KNN), Back propagation neural network (BPNN). Ten-fold cross validation was used for testing the trained classifiers. The overall classification accuracy (sensitivity) was defined as the number of true positives divided by the sum of true positives and false negatives. Our datasets were imbalanced since cells are in interphase most of the time (58% or higher) (Figure 2). To overcome the bias towards the class with the highest frequency, we adopted a weighted-SVM technique [21]. The weighted-SVM approach achieved the best classification result when the weight of each class was inversely proportional to the square root of the number of training samples in its class.

### Visualization and validation of classification outputs

**Figure 3:**
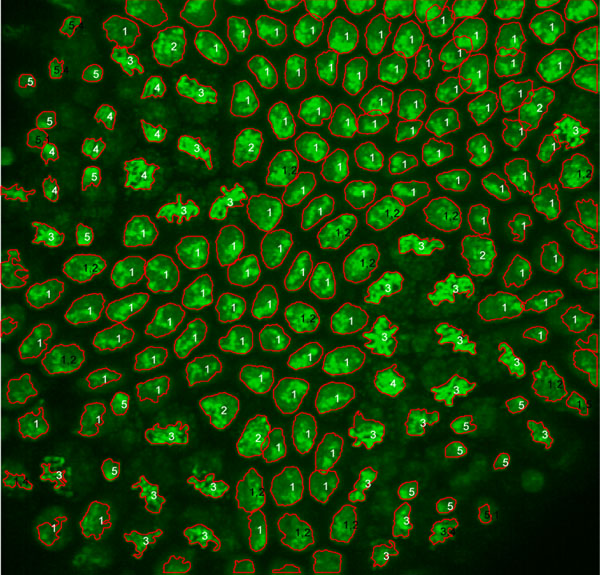
**Visualization and validation of classification outputs.** Silhouette contours (red) are superimposed on the maximum intensity projection of a mitotic domain in the head region of a *Drosophila* embryo during gastrulation. Correct class predictions are indicated in white and classification errors in black letters (predicted, actual class). 1=interphase, 2=prophase, 3=metaphase, 4=anaphase, 5=telophase.

To validate the classification results, we designed a visualization tool. The classification results are superimposed on maximum intensity projections (MIP) of 3D image stacks together with the ground truth labelling by the human expert (Figure 3). If the classification result agrees with ground truth labelling, the label will be shown in white color, otherwise both of them will be shown in black color. The contour of each nucleus is drawn in red color, allowing the user to relate segmentation quality to classification performance.

## Results

We created two datasets of nuclei detected in 3D images of early *Drosophila* embryos labelled with the live reporter histone-GFP that visualizes the progression through the phases of the division cycle (Figure 1). The first dataset contained 4606 samples in various phases of nuclear divisions during in the syncytial blastoderm stage, while the second one contained samples of nuclei in proliferating epithelial cells during gastrulation. Syncytial blastoderm and gastrulation are separated by cellularization that lasts approximately one hour. For each sample, we calculated 42 intensity, shape and texture features and assigned the respective cell cycle phase; interphase, prophase, metaphase, anaphase or telophase.

The performance of different combinations of feature reduction and machine learning techniques were evaluated using the post-cellular blastoderm dataset (Table 1). The dimensionality after feature reduction was set to 8 for PCA and MDS, which is estimated from the intrinsic dimensionality of original data, and 4 for LDA, which is limited by the number of classes [22,23]. All parameters were tuned for optimal classification accuracy. The best performance of KNN was achieved when K was set to 10. For BPNN, 25 nodes were used in the hidden layer. We used C-SVM from the lib-SVM library [20]. The gamma and cost for the SVM were set to 0.001953 and 512, respectively. SVM outperformed other methods. Feature reduction techniques (PCA, LDA and MDS) did not improve classification accuracy significantly.

**Table 1:** Comparison of cell cycle phase classification accuracy obtained with different classification models (columns) and feature reduction techniques (rows).

Accuracy	SVM	PNN	KNN	BPNN
Original features	93.52±0.62%	91.67±0.69%	90.18±0.56%	89.97±0.65%
PCA	92.45±0.73%	90.12±0.67%	90.02±0.54%	89.82±0.64%
LDA	93.12±0.45%	89.94±0.70%	89.12±0.56%	88.54±0.54%
MDS	93.23±0.44%	91.12±0.56%	91.34±0.65%	90.22±0.65%

The training set consisted of 3119 samples in 5 cell cycle phases (see breakdown in Table 2) of the post-cellular blastoderm (gastrulation). Training was performed using 10 fold cross validation. SVM=support vector machine, PNN= probabilistic neural network, KNN=k nearest neighbour, BPNN=neural network with back propagation, PCA=principal component analysis, LDA=linear discriminant analysis, MDS=multidimensional scaling.

**Figure 4:**
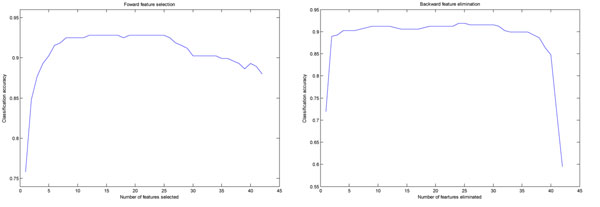
**Feature selection and cell phase classification accuracy.** (a) In forward feature selection, features were added one at a time according to importance. (b) In backward feature elimination, features were el iminated one at a time starting from the original set of 42.

**Figure 5:**
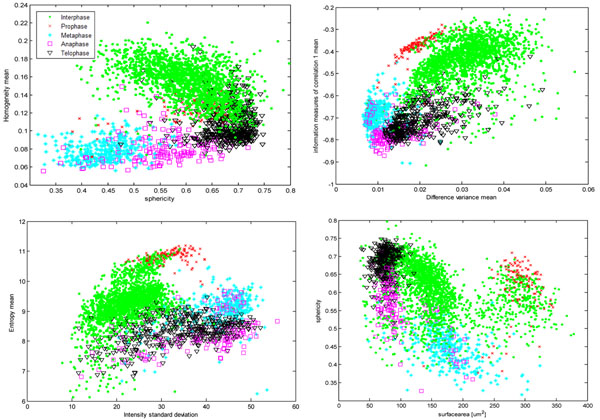
Scatterplots of the 8 dominant features.

We also used forward selection and backward elimination techniques to identify the dominant among the initial 42 features [19] (Figure 4). We used PNN for feature selection, as other classifiers require repeated parameter tuning for every new combination of features. After forward selection, we achieved the highest classification accuracy of 92.83% when we used the following 12 dominant features: 3 shape (sphericity, eccentricity E1, volume), 2 intensity (deviation between intensity-weighted and geometrical centroids in z, intensity standard deviation) and 7 texture features (mean homogeneity mean, mean information measures of correlation, difference variance mean, entropy mean, sum entropy mean, energy standard deviation, cluster shade standard deviation). Using backward elimination, we identified the following 12 features that achieved a classification accuracy of 92.18%: 2 were related to shape (eccentricity E1, sphericity), 1 to intensity (deviation between intensity-weighted and geometrical centroids on z direction) and 9 to texture (homogeneity standard deviation, cluster shade mean, sum variance mean, cluster shade standard deviation, variance standard deviation, difference entropy standard deviation, contrast mean, information measures of correlation 1 mean, information measures of correlation 2 standard deviation). Based on the feature selection results and exploratory data analysis (Figure 5), we selected the following 9 features for subsequent classifier training: sphericity, surface area, homogeneity mean, information measure of correlation 1 mean, difference variance mean, entropy mean, intensity standard deviation and deviation between intensity-weighted and geometrical centroids in the z direction.

Classification performance varied between cell cycle phases (Table 2), ranging from 66% for anaphase to 97% for interphase. The heterogeneity in prediction accuracy could be due to an imbalance in training dataset. Mitosis occupies a relatively short period of the cell cycle. Hence, interphase is predominant, while prophase and anaphase only represent 4% and 5% of total population. Using weighted-SVM, significantly improved the classification accuracy of prophase and telophase to 90.07% and 83.40%, respectively (Table 3). We also determined classification performance of the weighted SVM applied to a second dataset containing 4606 nuclei of the syncytial blastoderm stage. We observed an overall classification accuracy of 92.40% (Table 4).

**Table 2:** Cell cycle classification accuracy for a dataset of 3119 samples derived from the gastrulation blastoderm stage using none-weighted SVM and 42 features. (Pred. = predicted)

	Pred. Inter	Pred. Pro	Pred. Meta	Pred. Ana	Pred. Telo	True Total	Accuracy
True Inter	2002	16	12	2	31	2063	97.04%
True Pro	17	95	7	0	0	119	79.83%
True Meta	3	4	304	13	1	325	93.53%
True Ana	6	0	30	111	20	167	66.46%
True Telo	42	0	0	7	396	445	88.99%
Pred. Total	2070	115	353	133	448	3119	93.52%

**Table 3:** Cell cycle classification accuracy for a dataset of 3119 samples derived from the gastrulation stage using weighted-SVM and 9 features. (Pred. = predicted)

	Pred. Inter	Pred. Pro	Pred. Meta	Pred. Ana	Pred. Telo	True Total	Accuracy
True Inter	1877	54	7	14	111	2063	90.99%
True Pro	3	108	8	0	0	119	90.75%
True Meta	0	5	293	27	0	325	90.15%
True Ana	1	0	16	140	10	167	83.83%
True Telo	13	0	0	34	398	445	89.44%
Pred. Total	1894	167	324	215	519	3119	90.29%

**Table 4:** Cell cycle classification accuracy for a dataset of 4606 samples derived from the syncytial blastoderm stage using weighted-SVM and 9 features. (Pred. = predicted)

	Pred. Inter	Pred. Pro	Pred. Meta	Pred. Ana	Pred. Telo	True Total	Accuracy
True Inter	2293	87	3	4	59	2446	93.7%
True Pro	20	306	32	0	1	359	85.24%
True Meta	7	20	755	19	0	801	94.26%
True Ana	5	0	32	313	26	376	83.24%
True Telo	15	0	1	19	589	624	94.39%
Pred. Total	2340	413	823	355	675	4606	92.40%

As development progresses from the syncytial blastoderm to gastrulation, nuclei are encapsulated into a cell membrane. Upon cellularization, nuclei in epithelial cells elongate along the apical basal axis, leading to a change of nuclear shape from round to oval. Despite the developmental changes, interphase nuclei and mitotic chromosomes have a similar appearance in syncytial blastoderm and gastrulation. To test if cellularization and differentiation change sample distribution in the feature space we performed training and testing of weighted-SVM classifiers for samples from different developmental stages (Table 5). Cell cycle phase prediction of gastrula samples decreased from 90% to 50% when we used them as inputs for a SVM trained using syncytial blastoderm samples compared to a classifier trained for the same stage. In the complementary experiment, the prediction of syncytial blastoderm samples decreased from 92% to 70%. When we combined samples from both datasets we obtained a more robust classifier that could predict samples from both developmental stages at over 90% accuracy.

**Table 5:** Cell cycle phase classification performance for different training and testing datasets. We used a weighted SVM with 9 features.

Training	syncytium	gastrulation	syncytium	gastrulation	syncytium + gastrulation	syncytium + gastrulation
Testing	syncytium	gastrulation	gastrulation	syncytium	gastrulation	syncytium
Inter	93.7%	90.99%	52.93%	88.21%	93.46%	89.44%
Pro	85.24%	90.75%	6.67%	51.52%	91.60%	90.57%
Meta	94.26%	90.15%	46.33%	31.94%	90.78%	92.53%
Ana	83.24%	83.83%	44.07%	80.23%	80.01%	92.87%
Telo	94.39%	89.44%	69.83%	57.95%	86.52%	90.58%
Total	92.40%	90.29%	51.65%	70.52%	91.38%	90.08%

## Discussion

We noticed that a large proportion of misclassified cells were wrongly predicted to belong to neighbouring classes (see confusion matrices in Tables 2,3,4). For instance, 16 anaphase samples were misclassified as metaphase, and 10 anaphase samples as telophase (Table 3). This is not unexpected as phenotypic transitions of chromosomes during cell cycle progression happen gradually and there are no clear morphological boundaries between mitotic phases. Both forward feature selection and backward feature reduction could reduce the feature set from 42 to 12 without compromising classification performance (Table 4). Feature selection had a slight advantage as it was computationally more efficient (~2 times faster).

Although nuclei at syncytial and gastrula stage are visually similar, the overall classification accuracy of syncytial samples applied to a model trained with gastrula data was only 51.65%, while 70.52% classification accuracy was achieved in the converse experiment (Table 5). This might due to the following 3 differences: first, they are at different developmental stages, nuclei in syncytium stage have no membranes; second, they are from different *Drosophila* embryos; third, the laser power and microscope settings might be different for these two datasets. The results indicate that classifiers trained using syncytium dataset cannot be used to classify cells at cellular blastoderm stage and vice versa. However, a unified classifier can be obtained when trained using combining datasets from two developmental stages. Using this unified classifier, we could achieve over 90% classification accuracy for both datasets as shown in the last two columns of Table 5. This result shows that if the classifier is trained using more training samples containing all possible variations, a robust classifier can be obtained.

3D image stacks obviously contain more information than 2D images. Therefore, it is conceivable that 3D possess a higher discrimination power than 2D features. Since this notion lacks thorough evaluation and computing 2D features (especially texture features) is computationally less costly, it is worthwhile to address this issue in future research. One approach could involve producing 2D projections of 3D objects and testing the classification performance using 2D features extracted from 2D projections. Alternatively, we could extract features from a single representative slice (e.g. middle) as previously described [7].

## Conclusion

3D live cell imaging is becoming a common technique for the study of dynamic cellular processes in 3D tissues. Accurate cell phase classification is one of the essential steps to automate 3D live cell imaging analysis. Starting from an initial set of 42 shape, intensity and texture feature, we evolved a reduced subset of 9 dominant features without affecting predictive performance. Weighted-SVM was used to alleviate the problem of imbalanced training datasets. Over 90% classification accuracy was achieved on two dataset consisting of over 7000 cells (nuclei). As in cultured cells, automated cell cycle classification in 3D tissues can be applied to the characterization of cell divisions phenotypes resulting from genetic perturbations in multi-cellular organisms such as *Drosophila*, zebrafish or *C. elegans*. Our method does not depend on dynamic features derived from cell tracking. As such, this approach can be used to improve the performance of automated cell tracking in live cell imaging.

## Authors' contributions

THD designed and implemented the feature extraction and classification methodology, performed data analysis and drafted the manuscript. WCP acquired the 3D images of live Drosophila embryo, performed image segmentation and labelled the training sets. MW directed the project, was involved in conceptual design, data interpretation and drafting of the manuscript. All authors have read and approved the final manuscript.

## Competing interests

The authors declare that they have no competing interests.
